# Halting Progression From Prediabetes to Diabetes Through Patient Education: A Quality Improvement Project in an Underserved Community

**DOI:** 10.7759/cureus.103158

**Published:** 2026-02-07

**Authors:** Dinara Salimova, Tatevik Aloyan, Syed Hamza Shah, Alisher Makhmutov, Chimamaka Ikoro, Alizay Khan, Mouaz Oudih, Sophio Kakabadze, Ricardo Loor

**Affiliations:** 1 Internal Medicine, Ascension Saint Joseph, Chicago, USA

**Keywords:** lifestyle modification​, pre-diabetes, prevention of diabetes, quality improvement project, underserved populations

## Abstract

Background: The incidence of prediabetes and diabetes increases each year drastically worldwide, including in the United States. Statistically, 5-10% of individuals with prediabetes will develop diabetes each year. Underserved populations are particularly vulnerable to prediabetes progression due to lower health literacy, language barriers, limited access to healthcare, and socioeconomic challenges. Lifestyle modifications can reduce progression from prediabetes to diabetes by at least 40%, according to the literature.

Objective: The goal of the study was to increase awareness of prediabetes among an underserved patient population attending a community health clinic and to assess changes in glycemic and anthropometric parameters following patient education.

Methods: A single-group, pre-post observational design was conducted as part of a quality improvement (QI) project at a community health clinic serving an underserved population in Chicago, Illinois. Patients were advised to follow up within a 3-6-month period for assessment of HbA1c, fasting glucose, and body mass index (BMI) changes.

Results: A total of 46 patients with prediabetes received education on prediabetes and lifestyle modifications. Of these, 25 patients completed both pre- and post-intervention glycemic laboratory and anthropometric assessments and were included in the final analysis. Following patient education, mean HbA1c decreased from 6.03 ± 0.22% at baseline to 5.88 ± 0.23% at 3-6 months post-intervention, corresponding to a mean reduction of -0.15% (paired t-test, p = 0.0005). Fasting blood glucose values before and after the intervention were available for 16 of the 25 participants. Mean fasting blood glucose decreased from 105 ± 19.0 mg/dL pre-intervention to 97 ± 8.6 mg/dL post-intervention, corresponding to a mean reduction of -8 mg/dL; however, the result did not reach statistical significance (paired t-test, p = 0.064). All 25 patients had pre- and post-intervention BMI recorded. Mean BMI decreased from 33 ± 8.8 kg/m² at baseline to 32.6 ± 8.6 kg/m² post-intervention (paired t-test, p = 0.022).

Conclusion: This quality improvement initiative demonstrates that a single educational session may modestly improve HbA1c levels among underserved patients with prediabetes, highlighting the importance of developing more structured and intensive interventions to prevent progression to type 2 diabetes in this high-risk population.

## Introduction

Prediabetes is defined as higher-than-normal glycemic values that have not yet reached the criteria for the diagnosis of diabetes. It is associated with disrupted insulin sensitivity and impaired beta cell function and concomitantly causes nephropathy, neuropathy, retinopathy, macro- and microvascular disease, which are traditionally thought to be complications of diabetes [1].

According to the CDC, an estimated 115.2 million adults aged 18 years or older had prediabetes in 2023. Depending on the degree of dysglycemia, from 5 to 10% of individuals will develop diabetes [2], while according to the American Diabetes Association (ADA), up to 70% of individuals will eventually have progression of prediabetes to diabetes in a lifetime period. Multiple trials and systematic reviews have shown a diabetes incidence risk reduction of at least 40% after the application of lifestyle modifications in patients with impaired glucose tolerance [3-6], making this measure a well-known and first-line implication for the prevention of prediabetes progression. 

Per the CDC report, only 21.4% of adults with prediabetes acknowledged that they were informed about their diagnosis by a health professional [7]. Given that about 80% of the population with impaired glucose metabolism are not aware of their diagnosis, it becomes crucial to increase population awareness of prediabetes and measures to prevent it. The underserved population becomes the most vulnerable due to limited access to health care.

## Materials and methods

This study employed a single-group, pre-post observational design as part of a quality improvement (QI) project conducted at a CommunityHealth serving an underserved population in Chicago, Illinois. This project was reviewed by the clinic administration and determined to meet criteria for a quality improvement initiative and was exempt from institutional board review. 

Participants were adults aged 18 years or older with prediabetes, defined as a hemoglobin A1c (HbA1c) level between 5.7% and 6.4%, who attended the clinic between June 2025 and December 2025. Only patients with available pre- and post-intervention laboratory values were included in the analysis. Patients with a prior diagnosis of diabetes mellitus were excluded. Individuals receiving glucose-lowering medications, including metformin, sodium-glucose cotransporter 2 (SGLT2) inhibitors, glucagon-like peptide-1 (GLP-1) receptor agonists, or other antihyperglycemic agents, were excluded. Additional exclusion criteria included known chronic kidney disease and hemoglobin levels below 10 g/dL or above 18 g/dL.

The intervention consisted of a single educational session designed to increase patient awareness of prediabetes and promote lifestyle modifications aimed at reducing progression to diabetes. Education focused on dietary changes, physical activity, weight management, and the importance of regular follow-up. Educational content was delivered verbally and supplemented with written materials in English, Spanish, or Polish. The educational flier (provided in the Appendix; English version, pages 1-4) was developed by CommunityHealth based on CDC recommendations and distributed during routine primary care visits.

Clinical and laboratory data were extracted from the electronic medical record system (athenaOne [athenahealth, Boston, Massachusetts]). Variables collected included HbA1c, fasting blood glucose, and BMI at baseline and at follow-up visits. Patients were advised to return for follow-up at either 3 or 6 month based on availability and clinical considerations. Baseline medication use, including statins, thiazide diuretics, and corticosteroids, was also recorded.

The primary outcome was the change in HbA1c from baseline to post-intervention follow-up. Secondary outcomes included changes in fasting blood glucose and BMI, as well as the proportion of patients achieving normoglycemia, defined as an HbA1c level below 5.7%.

Pre- and post-intervention outcomes were compared using paired t-tests. Continuous variables were summarized using means and standard deviations. A two-tailed p-value < 0.05 was considered statistically significant. Statistical analyses were performed using Microsoft Excel and Google Sheets.

## Results

A total of 46 patients with prediabetes received education on prediabetes and lifestyle modifications. Of these, 21 patients (46%) did not complete follow-up within the 3-6-month period. The remaining 25 patients completed both pre- and post-intervention glycemic laboratory and anthropometric assessments and were included in the final analysis. 

**Figure 1:**
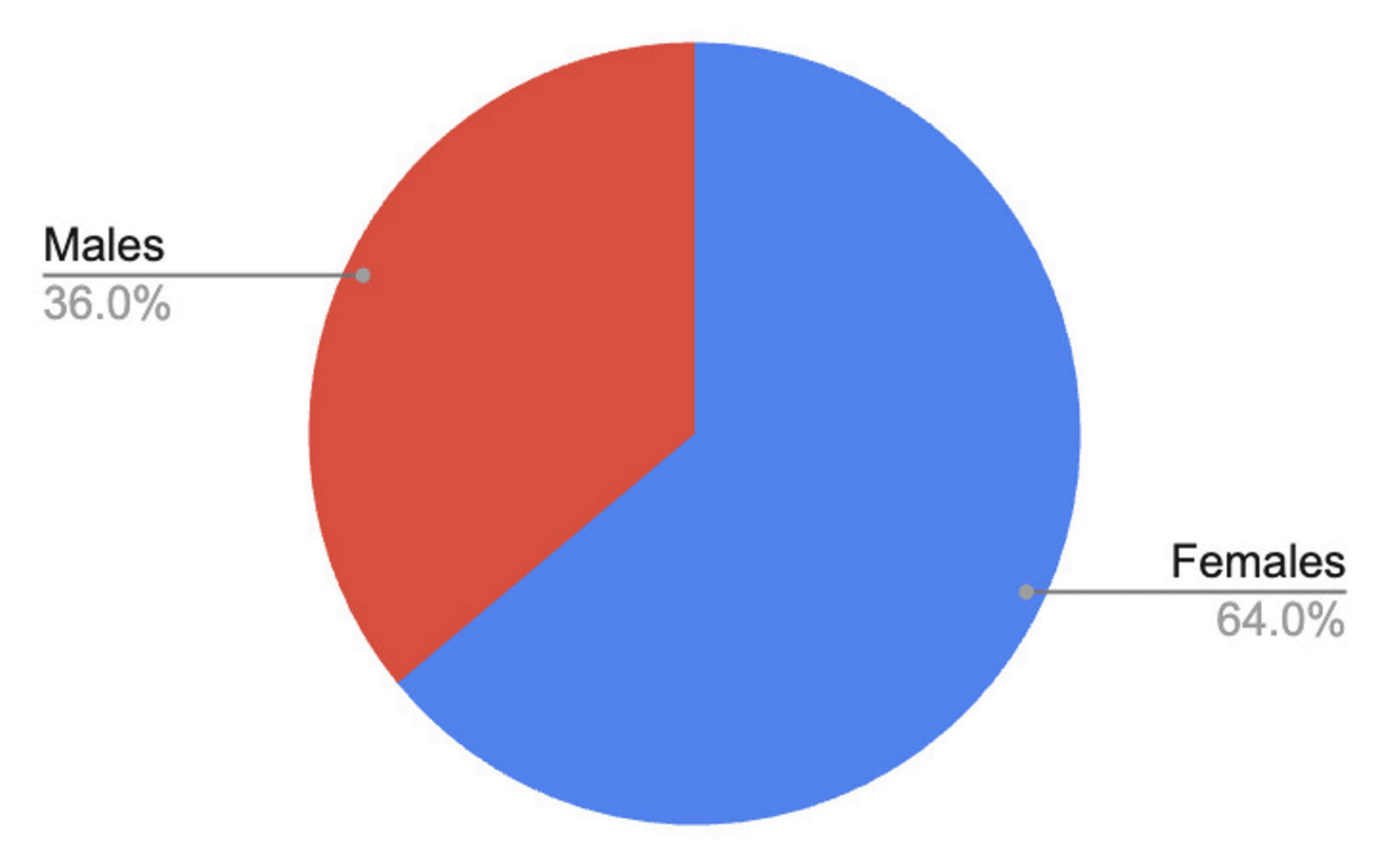
Gender distribution of the study population

**Figure 2:**
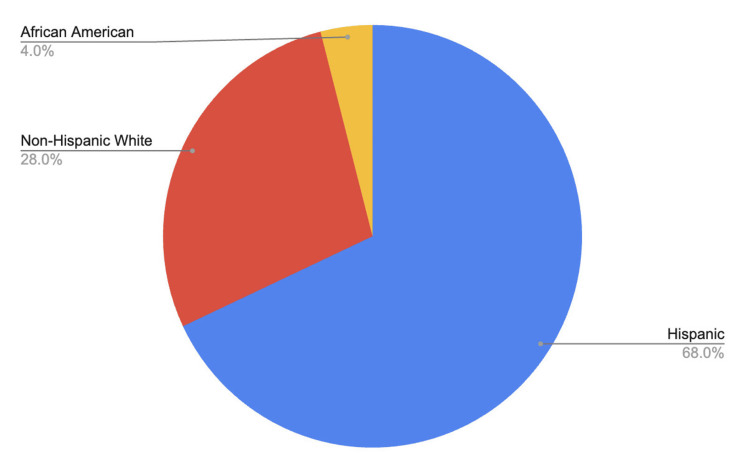
Ethnic distribution of the study population

Participant age ranged from 30 to 71 years old, with a mean age of 39.6 years. The study population included 16 females (64%) and 9 males (36%) (Figure 1). Ethnic composition consisted of 17 Hispanic participants (68%), 7 non-Hispanic White participants (28%), and an African American participant (4%) (Figure 2). Baseline medication use included statins in seven patients (28%), topical or inhaled corticosteroids as needed in three patients (12%), and thiazide diuretics in two patients (8%). 

**Figure 3:**
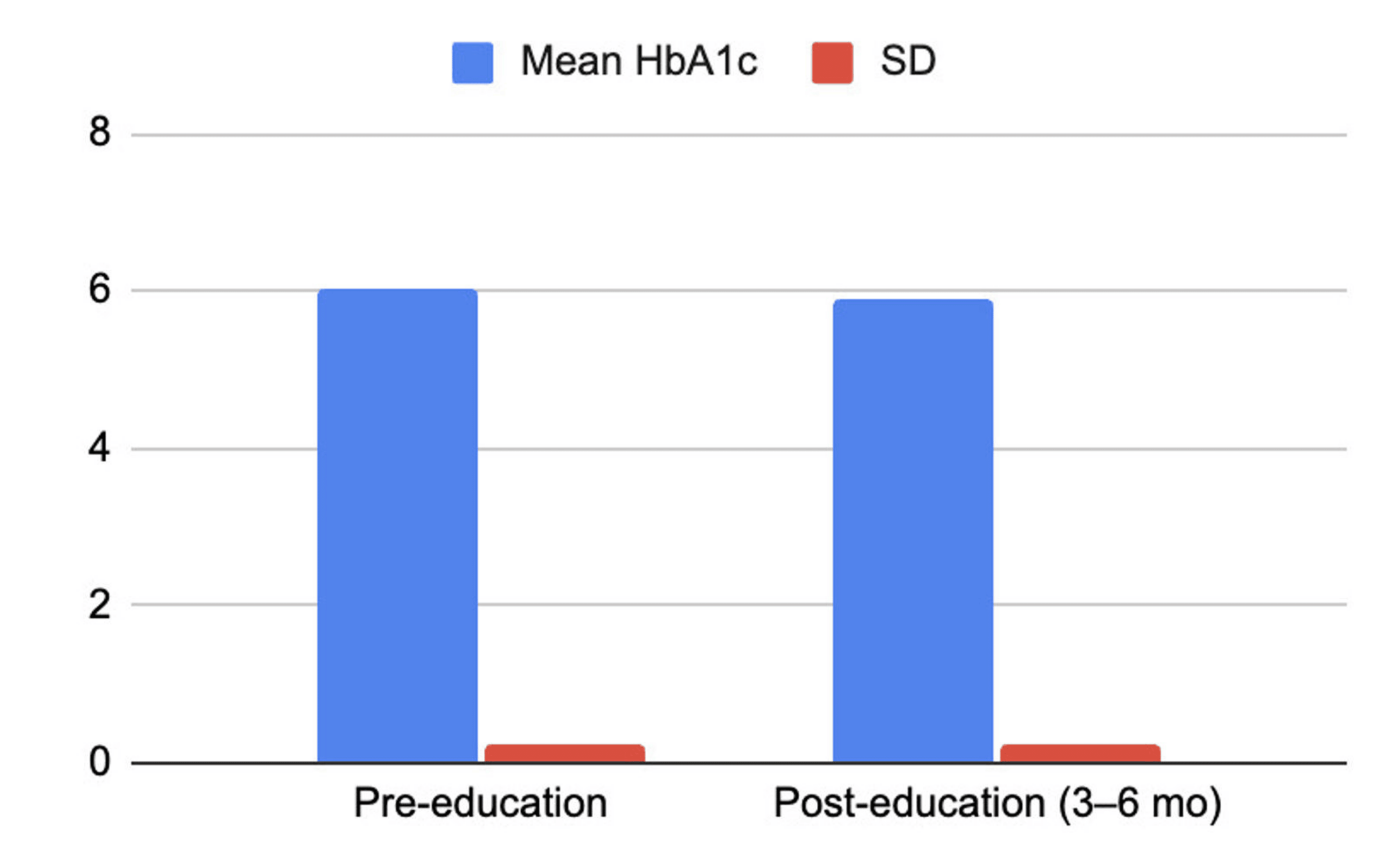
Degree of HbA1c change observed 3-6 months after the educational intervention Mean HbA1c decreased from 6.03% (SD 0.22) pre-education to 5.88% (SD 0.23) at 3–6 months post-intervention (mean change: −0.15 percentage points). HbA1c: Hemoglobin A1c, SD: standard deviation

**Figure 4:**
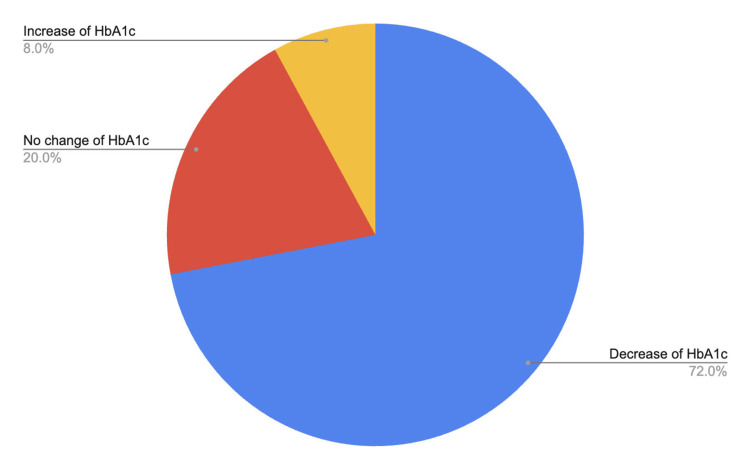
Distribution of post-education change in HbA1c levels

Following patient education, mean HbA1c decreased from 6.03 ± 0.22 at baseline to 5.88 ± 0.23 at 3-6 months post-intervention, corresponding to a mean reduction of -0.15% (Figure 3). This difference was statistically significant (paired t-test, p = 0.0005). HbA1c decreased in 18 of 25 patients (72%), remained unchanged in five patients (20%), and increased in two patients (8%) (Figure 4). Among patients demonstrating HbA1c reduction, follow-up measurements were obtained at six months in 11 patients, three months in four patients, five months in one patient, and four months in two patients. Two patients (8%) achieved normalization of HbA1c levels within the follow-up period.

Fasting blood glucose values before and after the intervention were available for 16 of the 25 participants, as nine patients did not fast before laboratory testing. Mean fasting blood glucose decreased from 105 ± 19.0 mg/dL pre-intervention to 97 ± 8.6 post-intervention, corresponding to a mean reduction of -8 mg/dL; however, this change did not reach statistical significance (paired t-test, p = 0.064). 

All 25 patients had pre- and post-intervention body mass index (BMI) recorded. Mean BMI decreased from 33 ± 8.8 kg/m2 at baseline to 32.6 ± 8.6 kg/m2 post-intervention. Although this reduction was statistically significant (paired t-test, p = 0.022), the magnitude of change was small.

## Discussion

It is a continuous process of altered glucose homeostasis that leads to the development of type 2 diabetes from normal glucose tolerance through progressive disruption of insulin sensitivity and impaired beta-cell function. Longitudinal studies, including the Whitehall II study, indicate that alterations in glucose regulation and beta-cell function can begin more than a decade before diabetes onset, with steep changes occurring approximately 3-6 years prior to diagnosis. While values demonstrated linear disruptions initially, this was later followed by steep changes in all of the parameters approximately 3 to 6 years prior to the diagnosis, supporting a multistage character of diabetes onset [1,8]. This suggests that prediabetes is a period during which glucose dysregulation is already accelerating, highlighting the potential value of early intervention to mitigate progression [8,9].

Extensive research has demonstrated that lifestyle interventions, including dietary modifications and increased physical activity, can reduce the risk of diabetes in individuals with prediabetes. A systematic review of 53 studies by Balk et al. reported that such interventions reduced diabetes incidence by at least 40% compared to usual care in individuals at risk of developing diabetes (including those with prediabetes). In addition to that, benefits of improved cardiometabolic risk profiles, weight loss, and, in some cases, reversion to normoglycemia were observed as well [5-6,10-11].

Underserved populations are particularly vulnerable to prediabetes progression due to lower health literacy, language barriers, limited access to healthcare, and socioeconomic challenges. Evidence indicates that lifetime risk of prediabetes and diabetes is higher among African American and Hispanic populations and that a substantial proportion of affected individuals remain undiagnosed or fail to engage in recommended interventions [12,13].

Patients eligible for care at the CommunityHealth are uninsured and have household incomes at or below 300% of the Federal Poverty Guidelines. Unfortunately, educational level is not collected during new patient registration, and therefore, no data on participants’ education were available or assessed during visits.

While providing care for the underserved population in the CommunityHealth, it was noted that a substantial number of patients with prediabetes were not aware of their diagnosis, the risk for subsequent development of diabetes, and the ways of prevention. At our clinic, educational classes are available for patients with diagnosed diabetes; however, none are available for prediabetes. To improve patient awareness of prediabetes and provide education regarding dietary and physical activity modifications to reduce its progression, we decided to conduct this quality improvement project.

In this quality improvement project, a single educational session on prediabetes and lifestyle modifications was delivered to patients in the underserved population. Post-intervention, we observed a statistically significant reduction in HbA1c, with a mean decrease of 0.15% over 3-6 months. The reduction was greater among patients with follow-up at six months compared to shorter intervals. Meta-analysis by Huang et al. demonstrated a 0.6-0.7% HbA1c reduction in prediabetic individuals. Although the magnitude of change was smaller than reported in previous studies, our results are consistent with the literature in showing that even limited education can produce measurable improvements [14,15]. The smaller effect in our project likely reflects the one-time nature of the intervention and the lack of structured monitoring of adherence to lifestyle recommendations.

These findings underscore that even a single educational intervention is beneficial in increasing patient awareness and improving glycemic markers, particularly in vulnerable populations at high risk for diabetes. However, our results also indicate the need for more intensive and sustained interventions, including multiple education sessions, ongoing support for lifestyle changes, and structured follow-up, to achieve larger reductions in diabetes risk.

This study has several limitations. Follow-up intervals were variable (3-6 months), and the sample size was small. A large number of patients were lost to follow-up. Potential confounders, including the use of statins, corticosteroids, or thiazide diuretics, were not controlled. There was no control group, as the study was a single-group, pre- and post-observational design, and the delivery of education may have varied between providers. Only a single educational session was provided; a structured educational program was not applied due to limited local resources at CommunityHealth. Adherence to recommended lifestyle modifications was not monitored or directly measured. Attrition bias may have affected the results, as patients who returned for follow-up were likely more motivated, potentially leading to an overestimation of the observed benefit. Educational level, a known determinant of engagement and effectiveness in community-based health education programs, was not assessed because it is not collected during new patient registration at CommunityHealth. The absence of these variable limits interpretation of the findings and precludes adjustment for its potential confounding effect. Finally, the short follow-up period limits the assessment of long-term outcomes.

## Conclusions

In conclusion, this quality improvement initiative demonstrates that a single educational session may modestly improve HbA1c among underserved patients with prediabetes and highlights the importance of developing more structured and intensive interventions to prevent progression to type 2 diabetes in this high-risk population.

## Figures and Tables

**Figure 5:**
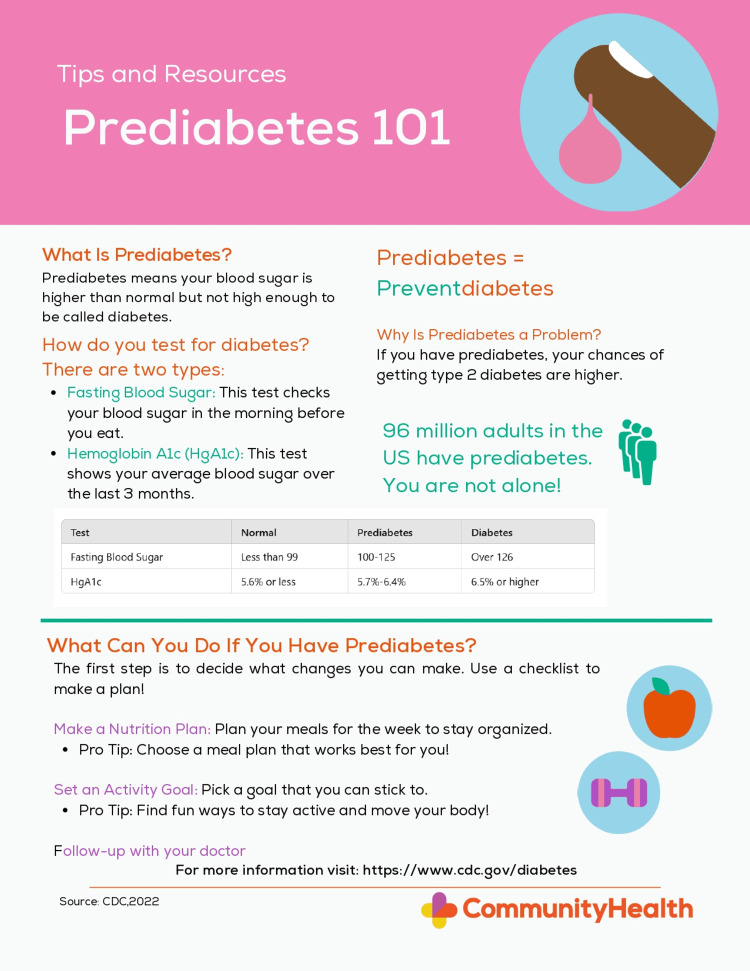
Prediabetes patient education (flier in English) - page 1

**Figure 6:**
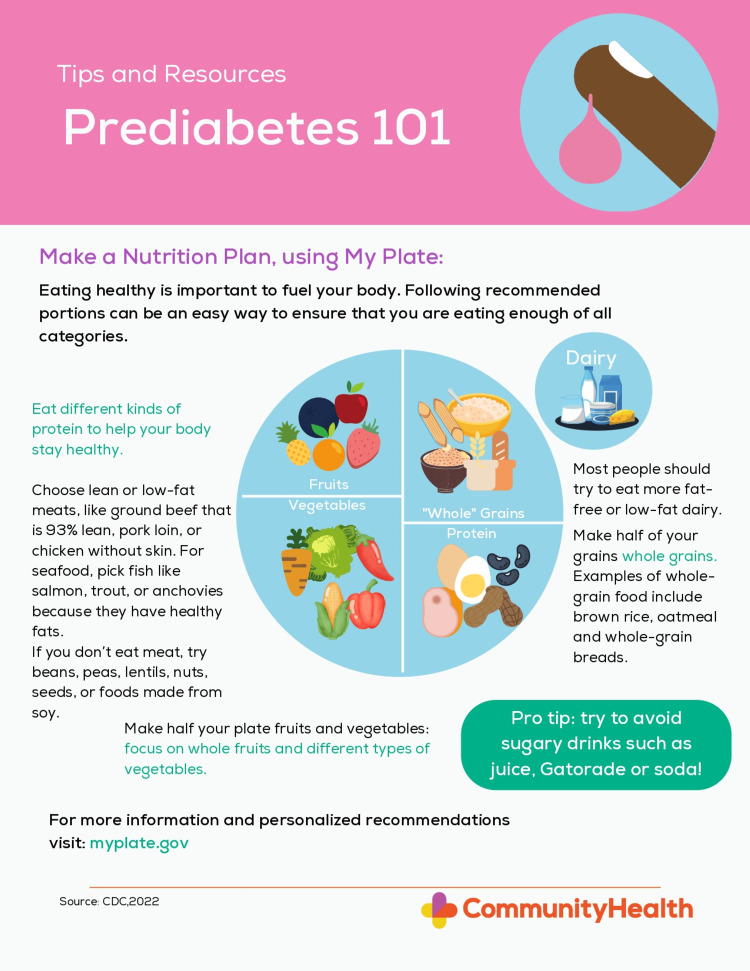
Prediabetes patient education (flier in English) - page 2

**Figure 7:**
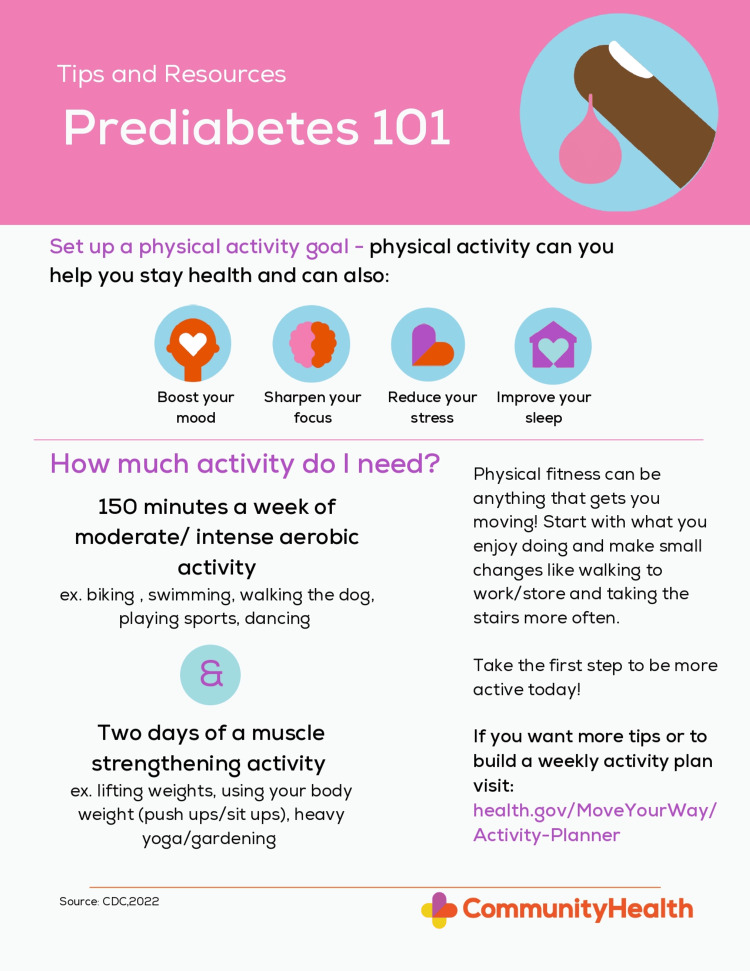
Prediabetes patient education (flier in English) - page 3

**Figure 8:**
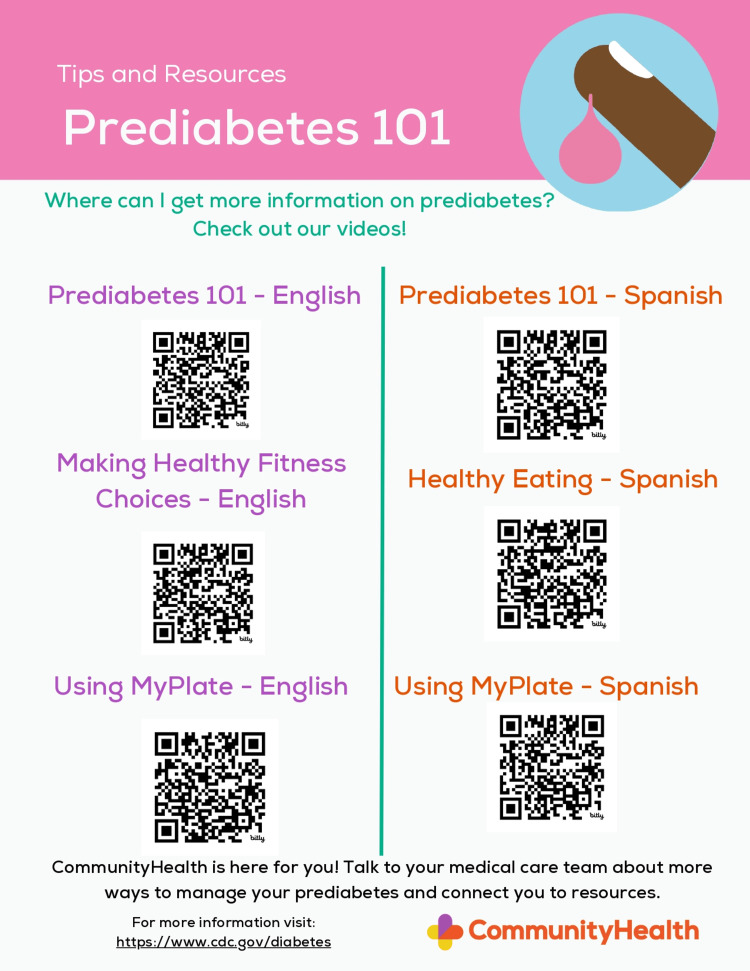
Prediabetes patient education (flier in English) - page 4
